# Red pepper powder is a crucial factor that influences the ontogeny of *Weissella cibaria* during kimchi fermentation

**DOI:** 10.1038/srep28232

**Published:** 2016-06-17

**Authors:** Bo Kyoung Kang, Min Seok Cho, Dong Suk Park

**Affiliations:** 1 Department of Agricultural Biotechnology, National Academy of Agricultural Science, Rural Development Administration, Jeonju, 54874, Republic of Korea

## Abstract

*Weissella cibaria* has been found in Korean kimchi and other sources, including fermented foods, Greek salami, Spanish sausages, and animal and human excrement. *W. cibaria* was recently reported to show anticancer, immunomodulatory, anti-inflammatory and antioxidant properties. Nevertheless, fundamental ecological succession studies are required to scientifically confirm the probiotic action of *W. cibaria* under various conditions, such as fermentation. Therefore, in the present study, we mined the *W. cibaria* KACC11862 genome in search of species-specific genes to use as new PCR targets for the detection and quantification of *W. cibaria* in kimchi. The sensitivity and specificity of the identified primer set from the putative outer membrane protein gene for the detection of *W. cibaria* KACC11862 in kimchi were analysed. Primer set specificity was evaluated using genomic DNA from eight *W. cibaria* isolates, 10 different species of *Weissella* and 13 other reference lactic acid bacteria (LAB) strains. Interestingly, by using the qPCR assay developed herein, we found that red pepper powder markedly affects the ontogeny of *W. cibaria* during kimchi fermentation.

*Weissella cibaria* is a Gram-positive, non-pore-forming, non-motile, hetero lactic acid-fermenting, and catalase-negative bacillus that cannot produce dextran from sucrose^1^,^2^. It has been found in Korean kimchi and has been isolated from various other sources, including fermented foods, Spanish sausages, Greek salami and human and animal excrement^3^,^4^,^5^. Recently, certain members of the *Weissella* genus, such as *W. cibaria*, *W. koreensis* and *W. confusa*, were reported to dominate during the early stage of kimchi fermentation. The proportion of *Weissella* was higher in kimchi with red pepper powder than in kimchi without red pepper powder, while the amounts of *Leuconostoc* and *Lactobacillus* were lower in kimchi with red pepper powder^6^. In addition, it was observed that red pepper powder strongly influenced the density of *W. koreensis*, which was associated with the anti-obesity effect of fermented kimchi^7^.

Kimchi is a naturally fermented vegetable food that has become emblematic of Korean culture. The most common type of whole kimchi (baechu-kimchi) is made by mixing salted white cabbage with a kimchi paste composed of red pepper powder (*Capsicum annuum*), Korean radish ginger, garlic, spring onion, fish sauce, starch paste and other ingredients, such as fresh seafood. White kimchi and watery kimchi are made without red pepper powder^7^. Kimchi consumption has a range of health benefits. Indeed, kimchi was shown to be effective against cancer, obesity, constipation and high cholesterol. Furthermore, kimchi also possesses fibrolytic, anti-oxidative and anti-ageing properties^8^. Previously, numerous studies on kimchi microbiology have identified three major genera of lactic acid bacteria (LAB), namely *Leuconostoc, Lactobacillus* and *Weissella*, that dominate in the finished product^6^. Recently, among these dominant genera, *W. cibaria* was reported to possess anticancer, anti-inflammatory, antibacterial, anti-fungal and immune-boosting potential^9^ because it induces higher levels of nitric oxide, nuclear factor (NF)-κB and cytokines (e.g., interleukin-1β and tumour necrosis factor-α). In addition, *W. cibaria* possesses an antiviral function against the avian influenza virus and was found to extend the lifespan of *Caenorhabditis elegans*^3^,^8^,^10^. Nevertheless, few unequivocal answers exist regarding the most fundamental properties of *W. cibaria* under conditions such as fermentation^7^,^11^. Indeed, only a few genomic analyses have been performed on strains in the *Weissella* genus, and the sequencing data available in public databases cover only a small number of strains. While there are currently 20 recognised species within this genus, the genomic data examined here represent only six species^12^. Therefore, we established an accurate, sensitive and practical SYBR Green quantitative polymerase chain reaction (qPCR) method to specifically monitor and quantify *W. cibaria* in diverse fermented foods. Currently, species-, subspecies- or strain-specific DNA probes are widely used to screen, detect, quantify and identify fungi, bacteria, yeast and viruses^13^. Numerous molecular assays that are based on the use of 16S rDNA together with a well-characterised metabolic gene are commonly used for the detection and identification of *Weissella* species. However, serious drawbacks have been observed for the specific identification and detection of *W. cibaria* isolates, as these assays also detect other *Weissella* species^14^. Additionally, many multiplex PCR and chromogenic DNA microarray systems have been developed for the simultaneous amplification of several genes in a single assay. However, these approaches exhibit some limitations, as the detection of target cells in food samples or in mixtures that vary widely with respect to their ratios of bacterial species is difficult^14^. Consequently, detection specificity, which can be influenced both by the uniqueness of the sequence within the targeted microbial genome of interest and by the precise annealing of the primers and probe to their target, is crucial to the efficiency of any PCR detection method.

Over the past decade, considerable efforts have been made to sequence various LAB strains. The growing number of LAB genome sequences available in public databases, combined with diverse bioinformatics tools, has promoted the emergence of methods that are more reliable, faster and more cost-effective for bacterial identification in an extensive range of samples. Particularly, LAB genome databases have made it possible to distinguish specific LAB strains from closely related lineages and species groups. However, despite recent scientific progress with respect to LAB strains in the microbiology industry, current methods for detecting, identifying and quantifying specific LAB strains are still substantially limited. Consequently, in this study, we mined publicly available genome sequence information (ftp://ftp.ncbi.nlm.nih.gov/genbank/) to develop a SYBR Green real-time PCR assay for the precise detection and quantitation of *W. cibaria*. A pair of species-specific primers based on a single gene from the *W. cibaria* KACC 11862 genome sequence was designed.

Bacterial membranes are known to have diverse functions in transport and mitochondrial activities and in the biosynthesis of membranes, cell walls and cellular capsules; the specific functions of membranes depend on whether they are specialised or are simply non-specialised cytoplasmic membranes. Membrane fusion proteins are found only in the prokaryotic world and function in conjunction with different types of transport systems in Gram-positive and Gram-negative bacteria. Bacterial membrane proteins are diverse in structure and function and show a wide size distribution^15^.

In the present study, we establish a reliable and efficient procedure for the quantitative detection of *W. cibaria* in kimchi samples using the SYBR Green PCR technique. Our results reveal that this method can be used for the specific detection and quantification of *W. cibaria* in various products. Remarkably, using this real-time PCR assay, we find that red pepper powder critically affects the ontogeny of *W. cibaria* during kimchi fermentation.

## Results

### Genomic analysis and *in silico* specificity test of the designed primer set

Specific primers (termed WC207F and WC207R) were designed from the genomic sequence of corresponding to a putative outer membrane protein-encoding gene of *W. cibaria* using the Lasergene PrimerSelect tool (Table 1). Because the specificity of the WC207F/WC207R primer pair had not been previously evaluated, thorough two-level specificity testing was undertaken. First, the specificity of the WC207F/R primer set was confirmed by using the BLAST and e-PCR analysis programs (http://www.ncbi.nlm.nih.gov/). The BLAST nucleotide program was set to the nr parameter, in which all the GenBank, RefSeq Nucleotide, EMBL, DDBJ and PDB sequences are queried. Furthermore, to increase the range of our specificity analysis, the databases that we searched for unintended gene sequence matches were not limited by organism type. The BLASTn searches returned no significant match to the known reference sequences. The BLASTx results using the predicted protein sequence revealed that the most similar protein to our hypothetical protein was a *W. confusa* protein [identity = 28%, score = 277 bits (709), and expected = 7e–74]. In addition, the WC207F/R primer set specificity was tested using conventional PCR techniques. For this analysis, DNA samples from 31 different strains were used (Table 2). The expected PCR product, with a length of 207 bp, was observed in all PCR assays performed using genomic DNA from *W. cibaria* strains. In contrast, the WC207F/R primer pair did not amplify any product when genomic DNA from other species was used as the template (Table 2). These findings show that the primers designed in this study are specific for *W. cibaria* strains and are therefore suitable for their identification.

### Standard curves and melting temperature

**Figure 1:**
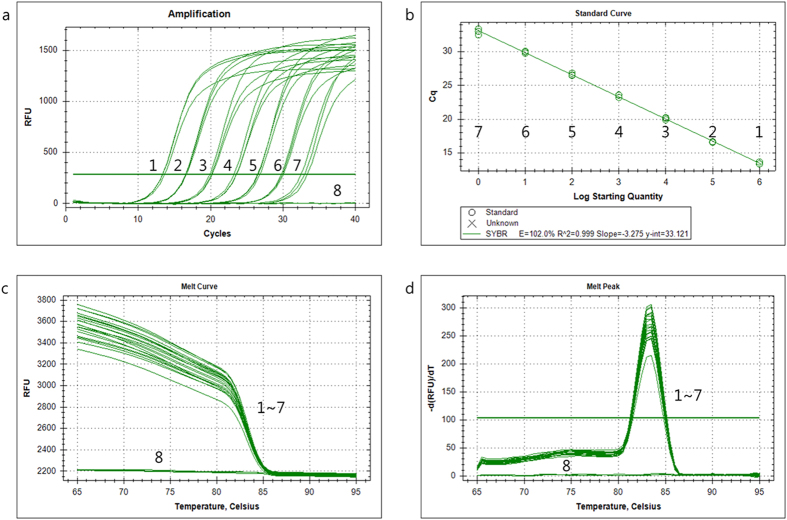
Specificity, melting peak and standard curve of the WC207F/R primer set using SYBR Green qPCR. (**a**) Fluorescence intensity as a function of the template concentration. For each assay, a series of 10-fold dilutions of cloned DNA (ranging from 1.44 × 10^3^ to 1.44 × 10^9^ copies/μl) were used as template (1–7, sample dilutions; 8, no-template control). (**b**) Standard curve derived from the amplification plot. (**c**) Melting curve analysis (1–7, sample dilutions; 8, no-template control). (**d**) Melting peak analysis (1–7, sample dilutions; 8, no-template control). The amplified products’ derivatives of relative fluorescence units [-d(RFU)/dT] were plotted as a function of temperature (amplified product, 83.0 °C). The high peak indicates the amplified product, and the low peak is the no-template control.

We used SYBR Green real-time PCR analysis to generate a standard curve with *W. cibaria* KACC 11862 by plotting the mean threshold cycle (Ct) (n = 3) based on the logarithmic concentrations of genomic DNA (ranging from 5 to 5 × 10^−5 ^ng/μl), cloned DNA (ranging from 1.44 × 10^9^ to 1.44 × 10^3^ copies/μl) and on the density of the cell suspension (ranging from 1.95 × 10^8^ to 1.95 × 10^5 ^CFU/ml) (Fig. 1a and Table 3). The limit of quantitation (LOQ) assay showed a good linear response and a high correlation coefficient (R^2^ = 0.999). A standard curve analysis of the linear portion of the slope resulted in a coefficient of −3.275, which yielded a PCR efficiency of 102.0% and a y-intercept value of 33.121 (Fig. 1b). Melting analysis (curve, temperature and peaks) with *W. cibaria* KACC 11862 produced from real-time PCR reactions reproducibly showed a melting temperature of 83.0 °C and specific peaks (Fig. 1c,d). The input genomic DNA concentration (R^2^ = 0.994, slope = −3.470) and the bacterial cell suspension (R^2^ = 0.975, slope = −2.902) standard curves exhibited linear correlations with their respective Ct values. The detection limits of the real-time PCR were 50 fg/μl and 1.95 × 10^5^ CFU/ml of reaction mix for the input genomic DNA concentration and the bacterial cell suspension of *W. cibaria* KACC11862, respectively (Table 3). The real-time PCR assay of *W. cibaria* KACC11862 displayed excellent quantification and accurate detection characteristics.

### Quantification of *W. cibaria* in kimchi samples using real-rime PCR

**Figure 2:**
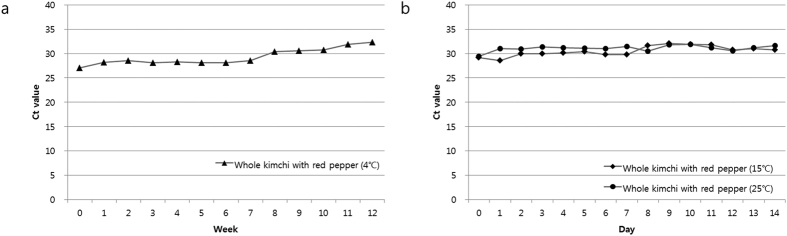
Changes in the real-time PCR Ct values during the quantification of *W. cibaria* from total DNA isolated from salted Chinese cabbage kimchi fermented at 4 °C (**a**), 15 °C or 25 °C (**b**).

Whole kimchi (Chinese cabbage fermented with salt and red pepper) and white kimchi (without red pepper powder) samples were purchased from the local market and evaluated. Two samples were stored at three different temperatures (4 °C, 15 °C and 25 °C) during the fermentation period. Each sample was examined for the presence of *W. cibaria* by SYBR Green real-time PCR. Importantly, regardless of the temperature and fermentation period, we observed a large difference in *W. cibaria* density between the two types of kimchi samples with respect to the presence or absence of red pepper powder. All of the samples taken from white kimchi and stored at 4 °C, 15 °C and 25 °C produced no fluorescence signal to indicate the Ct value, in contrast to the corresponding samples from whole kimchi (Fig. 2a). *W. cibaria* was also confirmed as a predominant psychrophilic species during the early fermentation stage of whole kimchi, but only at 4 °C; the lowest Ct values (reflecting the highest concentrations of starting DNA material) were measured between weeks 0 and 7, after which the Ct values increased gradually (Fig. 2b). In addition, the whole-kimchi samples that were stored at 15 °C and 25 °C showed very high Ct values and only minor changes in the density of *W. cibaria* (Fig. 2b). Consequently, we concluded that red pepper powder drastically influences the density of *W. cibaria* during kimchi fermentation, regardless of the temperature or fermentation period (Fig. 2).

## Discussion

Because of their ability to reach the gastrointestinal tract and to interact with the immune system of their host, probiotic bacteria are receiving substantial attention as vehicles for the delivery of biotherapeutics^16^. However, the study of probiotic communities currently faces a difficult roadblock^17^. Genome sequencing and functional genomics studies on a variety of LAB strains are now rapidly providing new insights into microbial community dynamics in diverse environmental samples, revealing the molecular basis for important traits such as sugar metabolism, stress responses, adaptation and interactions^18^. However, there is no clear evidence of the use of species-specific molecular probes to accurately quantify the proportion of a specific LAB in different ecosystems, preventing a complete understanding of how the abundance of a particular microbe that is associated with different types of food changes over time and how these changes affect the final quality of the fermented products.

Therefore, establishing a reliable and efficient process for the quantitative detection of specific LAB used in various lactic acid products is tremendously important, as it would enable the detection of specific species and overall community profile changes over time, at different temperatures, and under different conditions.

Early studies on kimchi LAB communities generally employed traditional, but often unsuccessful, methods based on the morphological and phenotypic identification of LAB species grown on agar culture media^6^. Indeed, this system has major drawbacks, such as a long assay time of 10 days and the detection of cultivable cells only. Consequently, molecular methods using the sequence of 16S ribosomal RNA and other genes for the identification of isolated strains have drawn the attention of many researchers^19^. However, these approaches are not appropriate for monitoring the succession of a targeted bacterial species during kimchi fermentation^19^. Therefore, the value of molecular detection and quantification methods for studying LAB is enormous.

Heretofore, all types of kimchi were thought to behave similarly to the kimchi samples that were used in previous studies. Specifically, an extensive diversity of bacteria was present at the beginning of the process; as fermentation progressed, the sample became dominated by three main genera, namely *Lactobacillus*, *Leuconostoc*, and *Weissella*^20^.

Red pepper powder in particular was shown to delay the kimchi fermentation progress, including LAB growth and metabolite production and especially during the early fermentation period. At the genus level, the proportion of *Weissella* was higher in kimchi with red pepper powder than in kimchi without red pepper powder, whereas the proportions of *Leuconostoc* and *Lactobacillus* were lower in kimchi with red pepper powder^20^. Recently, *W. koreensis*, which shows anti-obesity effects, was identified as the dominant species; it exhibits relatively good psychrophilic growth at 4 °C or colder. The density of *W. koreensis* is usually greater in whole kimchi with red pepper powder than in white kimchi without red pepper powder, regardless of the temperature and fermentation period. In addition, red pepper powder showed little influence on the cell proportions of *Lactobacillus plantarum* and *Leuconostoc mesenteroides* in kimchi, although temperature greatly impacted the density of *L. plantarum* (data not shown). Consequently, red pepper powder is estimated to greatly influence the proportion, or density of particular kimchi LAB strains^7^.

According to phylogenetic analysis based on 16S rRNA gene sequences, the genus *Weissella* was one of the predominant bacterial groups during kimchi fermentation^21^. Pyrosequencing analysis of commercial kimchi samples resulted in the genus *Weissella* being predominant at 44.4%. *W. koreensis* was 27.2% and *W. cibaria* was 8.7%^22^. However, the draft genome of *W. paramesenteroides* was a poor match to the metagenome sequence^21^. These results indicated that all *Weissella* species are not influenced by red pepper powder.

Generally, among the many bioactive materials found in kimchi, red pepper powder-derived capsaicin has been proposed as an effective source of anticancer, fat-digestion and diabetes-prevention properties^23^. However, little information exists on the correlation between red pepper powder and *W. cibaria* with respect to anticancer or fat-digestion effects.

Therefore, we identified species-specific genes using BLAST searches and developed a primer set to investigate these correlations. The species-specific primer set was designed using the *W. cibaria* KACC11862 (GenBank accession no. ZP_08416035.1, gi|332429607:82527-85805) whole-genome sequence. The outer membrane protein gene (GenBank accession no. ZP_08416035.1) was also confirmed to be highly variable among *Weissella* species.

Interestingly, in the present study, the presence of *W. cibaria* was clearly detected in whole kimchi with red pepper powder, but it was not detected at all in white kimchi without red pepper powder regardless of the temperature or fermentation period. In order to provide additional evidence that red pepper powder as a crucial factor that influences the ontogeny of *W. cibaria* during kimchi fermentation, we designed a new marker to detect novel gene of *W. paramesenteroides*. As a result, *W. paramesenteroides* was not detected in the whole kimchi or white kimchi regardless of red pepper powder (data not shown). Moreover, different kinds of kimchi were used to determine if the function of red pepper powder exists in other kimchi. *W. cibaria* was identified from the chonggak kimchi (with red pepper powder) during the early fermentation period, while it was not detected in the dongchimi and watery kimchi (without red pepper powder) (data not shown).

According to the previous report on kimchi metabolites to investigate the effect of red pepper on kimchi fermentation, the metabolite concentrations in kimchi with red pepper powder were a little higher than those in kimchi without red pepper powder, which was presumably due to the concentration effects of metabolites in kimchi supernatants influenced by the addition of red pepper powder. Therefore, it is thought that metabolites in kimchi were influenced by the addition of red pepper powder, hence having an influence on ontogeny of *W. cibaria* in whole kimchi^20^.

In addition, *W. cibaria* was more abundant at 4 °C than at 15 °C or 25 °C (Fig. 2). *W. cibaria* was confirmed as a relatively psychrophilic strain that predominates at 4 °C. However, the population of *W. cibaria* decreased rapidly from the middle of the fermentation period onwards. In general, the proportion of *W. cibaria*, whether incubated at 4 °C, 15 °C and 25 °C, was strikingly lower than that of *W. koreensis*^7^.

To control the ripening process in quality-controlled kimchi production, it is essential to accurately monitor changes in the microbial community *in situ* during the fermentation period^24^. The most important advantages of this newly developed assay are its specificity and rapidity. Indeed, it allows the species-specific identification and quantification of *W. cibaria* strains within an hour, without any prior cultivation step. The many replicates used for obtaining the standard curves and the small standard errors among these replicates demonstrated that the assay is reproducible and highly robust, even with DNA isolated from kimchi.

In conclusion, whether using DNA isolated from pure cultures or DNA mixtures extracted from kimchi, we show that this newly developed real-time PCR assay detects *W. cibaria* with high specificity and sensitivity and without any false-positive signals caused by other LAB strains. This real-time PCR assay may be a convenient method for the detection and quantification of *W. cibaria* in food for quality-management purposes.

## Methods

### Bacterial strains and DNA extraction

In total, 31 LAB strains were used to validate the specificity of the species-specific primers developed in this study (Table 2). Bacterial strains were obtained from the Korean Agricultural Culture Collection (KACC) and from the Belgian Coordinated Collections of Micro-Organisms (BCCM). All strains were routinely cultured at 30 °C for 2 days on de Man Rogosa Sharpe (MRS) agar (Oxoid, Basingstoke, Hampshire, UK). Total genomic DNA was extracted with a bacterial genomic DNA extraction kit from Qiagen (Hilden, Germany). The concentration and quality of the genomic DNA samples were verified using a NanoDrop^®^ ND-1000 Spectrophotometer (NanoDrop Technologies, Wilmington, DE, USA).

### Kimchi sampling

Two types of kimchi were purchased from a commercial factory in Korea. The 26 batches (13 batches each for two kinds of kimchi) were stored at 4 °C. In case of storage at 15 °C and 25 °C, 30 batches (15 batches each for two kinds of kimchi) were used, respectively. The kimchi samples used in the experiments were obtained on the same day that they were produced and were stored at different incubation temperatures (4 °C, 15 °C and 25 °C) until the sampling time. Periodically during the fermentation period, aliquots were filtered through four layers of coarse gauze (Daehan, Daejeon, Korea) to remove large kimchi particles, and the filtrates were pooled. The filtrates were centrifuged (13,000 rpm for 10 min at 4 °C) to harvest the microorganisms. One microliter of each sample (5 ng/μl) was centrifuged separately for quantitative analysis by real-time PCR. Total genomic DNA was extracted from the kimchi sample pellets according to a previously described procedure combining a bead-beating method with the Fast DNA Spin kit (MPbio)^25^.

### Genome analysis and primer design

The genome sequences from *W. cibaria* KACC 11862 and the other LAB strains used in this study were downloaded from the NCBI bacterial genome database (ftp://ftp.ncbi.nlm.nih.gov/genomes/bacteria/). We used bioinformatics sequence analysis technology to integrate the different steps and used a modified Chen and Lang tool for the candidate gene selection pipeline via computational clustering^26^,^27^. On the basis of these comparative outputs, target genes showing no significant concordance with the other LAB strains were selected as PCR targets. The primers (termed WC207F/R) used for *W. cibaria* KACC11862 (GenBank accession No. ZP_08416035.1) were designed using Lasergene PrimerSelect software (version 7.2.1; DNASTAR Inc., USA) on the putative outer membrane protein-encoding gene. These primers amplify a DNA fragment of 207 bp. The nucleotide sequences of the primers were assessed for specificity by using NCBI BLAST searches (http://blast.ncbi.nlm.nih.gov). The primers were synthesised by Bioneer Corporation (Daejeon, Korea) (Table 1).

### Conventional PCR

PCR amplifications were performed in a final reaction volume of 25 μl (1x buffer, 4.0 mM MgCl_2_, 0.2 mM each dNTP) containing 1.25 U of GoTaq^®^ Flexi DNA polymerase (Promega, Madison, WI, USA), a 0.2 μM final concentration of each primer (Table 1) and 25 ng of template DNA. The amplification conditions included an initial denaturation period of 5 min at 95 °C, 35 cycles of denaturation (1 min at 95 °C), annealing (30 s at 62 °C), and extension (1 min at 72 °C), and a final extension period of 7 min at 72 °C. All conventional PCR reactions were performed using a PTC-225 thermocycler (MJ Research, Watertown, MA, USA). The amplified products were stained with LoadingStar (DYNEBIO, Seoul, Korea) and analysed by electrophoresis using 1.5% (w/v) agarose gels. The bands were visualised using a VersaDoc 1000 gel imaging system (Bio-Rad Laboratories, Hercules, CA, USA).

### SYBR Green qPCR

The SYBR Green real-time PCR amplifications were performed using a CFX96 real-time PCR system (Bio-Rad Laboratories, Hercules, CA, USA) in a final reaction volume of 20 μl containing 5 ng of purified DNA from each sample, the abovementioned primers (0.5 μM final concentration) and iQ^TM^ SYBR^®^ Green Supermix (Bio-Rad Laboratories, Hercules, CA, USA). The real-time PCR amplifications were performed using the following cycling conditions: initial denaturation of 30 s at 95 °C, 40 cycles of 5 s at 95 °C and 30 s at 62 °C, and a melting curve analysis from 65 to 95 °C with an increment of 0.5 °C per 5 s. Analysis of the absolute quantification data was performed automatically using the Bio-Rad CFX Manager^TM^ Version 1.6 software package.

The LOQ and limit of detection (LOD) were determined as the amounts in 10-fold serial dilutions of plasmid DNA, genomic DNA or a bacterial cell suspension of *W. cibaria* KACC 11862 that corresponded to the threshold cycles at which the sum of the specificity and sensitivity of the assay was maximised. The quality of the SYBR Green real-time assay was determined by the absence of stochastic effects in the reaction efficiency and by whether or not nonspecific reaction products were formed.

The copy number of the template was calculated using the following formula^28^:


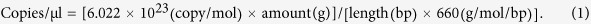


## Additional Information

**How to cite this article**: Kang, B. K. *et al*. Red pepper powder is a crucial factor that influences the ontogeny of *Weissella cibaria* during kimchi fermentation. *Sci. Rep.* **6**, 28232; doi: 10.1038/srep28232 (2016).

## Figures and Tables

**Table 1:** Primer sequences, their targets, and the annealing temperatures used in *W. cibaria* PCR screens.

Primer	Oligonucleotide sequence (5′-3′)	Annealing (°C)	Amplicon (bp)	Target gene	Reference
WC207F	TTG CGT TGT GAA ATC TGC T	62	207	putative outer membrane protein	This study
WC207R	GGT GCG GTC TTC CAA TAC T				

**Table 2:** Bacterial strains used in this study.

No.	Bacterial strains	Source	Origin	This study
1	*Weissella cibaria*	KACC 11862^T^	Chili bo	+ ^a^
2	*Weissella cibaria*	LMG 16479	Canary, liver	+
3	*Weissella cibaria*	LMG 18501	Human, gall	+
4	*Weissella cibaria*	LMG 18506	Cheese whey	+
5	*Weissella cibaria*	LMG 18507	Post-harvest deterioration of sugar cane	+
6	*Weissella cibaria*	LMG 18814	Dialysis patient, faeces	+
7	*Weissella cibaria*	LMG 21843	Partially fermented kimchi	+
8	*Weissella cibaria*	LMG 22786	Dog	+
9	*Weissella paramesenteroides*	KACC 11847^T^	Fermented sausages, dry salami	−
10	*Weissella koreensis*	KACC 11853^T^	Kimchi	−
11	*Weissella kandleri*	LMG 18979^T^	Desert spring	−
12	*Weissella viridescens*	KACC 11850^T^	Cured meat products	−
13	*Weissella minor*	KACC 13437^T^	Slime from milking machine	−
14	*Weissella soli*	KACC 11848^T^	Garden soil	−
15	*Weissella halotolerans*	KACC 11843^T^	Sausage	−
16	*Weissella confusa*	KACC 11841^T^	Saccharum officinarum	−
17	*Weissella hellenica*	KACC 11842^T^	Naturally fermented Greek sausage	−
18	*Weissella thailandensis*	KACC 11849^T^	Fermented fish	−
19	*Lactobacillus brevis*	LMG 6906^T^	Human, faeces	−
20	*Lactobacillus curvatus*	KACC 12415^T^	Milk	−
21	*Lactobacillus kimchii*	KACC 12383^T^	Kimchi	−
22	*Lactobacillus paraplantarum*	KACC 12373^T^	Beer	−
23	*Lactobacillus pentosus*	LMG 10755^T^	N.D.	−
24	*Lactobacillus plantarum*	LMG 6907^T^	Pickled cabbage	−
25	*Lactobacillus sakei*	KACC 12414^T^	Starter of sake (Moto)	−
26	*Leuconostoc carnosum*	KACC 12255^T^	Vacuum-packed beef stored at low temperature	−
27	*Leuconostoc citreum*	KACC 11860^T^	Honeydew of rye ear	−
28	*Leuconostoc gelidum*	KACC 12256^T^	Vacuum-packed meat	−
29	*Leuconostoc inhae*	KACC 12281^T^	Kimchi	−
30	*Leuconostoc lactis*	KACC 12305^T^	Milk	−
31	*Leuconostoc mesenteroides*	KACC 12312^T^	Fermenting olives	−

KACC, Korean Agricultural Culture Collection, Republic of Korea; LMG, The Belgian Co-ordinated Collections of Microorganisms (BCCM^TM^), Belgium. ^T^Type of strain. ^a^+, detected; −, not detected. N.D., not determined.

**Table 3:** Mean Ct end-point fluorescence of 10-fold serial dilutions of *W. cibaria* cloned DNA, genomic DNA and a cell suspension, as determined using real-time PCR.

Cloned DNA		Genomic DNA		Cell suspension	
Weight/μl reaction mix	Ct ± SD (*n* = 3)	Weight/μl reaction mix	Ct ± SD (*n* = 3)	CFU/ml reaction mix	Ct ± SD (*n* = 3)
5 ng (1.44 × 10^9 ^copies)	13.46 ± 0.19	N.D.^a^	N.D.	N.D.	N.D.
500 pg (1.44 × 10^8 ^copies)	16.58 ± 0.07	5 ng	17.29 ± 0.14	N.D.	N.D.
50 pg (1.44 × 10^7 ^copies)	20.08 ± 0.18	500 pg	20.53 ± 0.04	N.D.	N.D.
5 pg (1.44 × 10^6 ^copies)	23.45 ± 0.23	50 pg	24.07 ± 0.11	1.95 × 10^8^	24.36 ± 0.54
500 fg (1.44 × 10^5 ^copies)	26.62 ± 0.20	5 pg	27.39 ± 0.08	1.95 × 10^7^	27.87 ± 0.16
50 fg (1.44 × 10^4 ^copies)	29.93 ± 0.16	500 fg	30.45 ± 0.07	1.95 × 10^6^	30.97 ± 0.22
5 fg (1.44 × 10^3^ copies)	32.95 ± 0.46	50 fg	34.96 ± 1.16	1.95 × 10^5^	32.79 ± 0.22

^a^N.D., Not determined.
